# Genomic prediction of synthetic hexaploid wheat upon tetraploid *durum* and diploid *Aegilops* parental pools

**DOI:** 10.1002/tpg2.20464

**Published:** 2024-05-19

**Authors:** Susanne Dreisigacker, Johannes W. R. Martini, Jaime Cuevas, Paulino Pérez‐Rodríguez, Nerida Lozano‐Ramírez, Julio Huerta, Pawan Singh, Leonardo Crespo‐Herrera, Alison R. Bentley, Jose Crossa

**Affiliations:** ^1^ International Maize and Wheat Improvement Center (CIMMYT) Texcoco México; ^2^ Aardevo B.V. Nagele The Netherlands; ^3^ Universidad Autónoma del Estado de Quintana Roo Chetumal México; ^4^ Colegio de Postgraduados, Campus Montecillos Texcoco México; ^5^ Australian National University, Research School of Biology Canberra Australia

## Abstract

Bread wheat (*Triticum aestivum* L.) is a globally important food crop, which was domesticated about 8–10,000 years ago. Bread wheat is an allopolyploid, and it evolved from two hybridization events of three species. To widen the genetic base in breeding, bread wheat has been re‐synthesized by crossing durum wheat (*Triticum turgidum* ssp. *durum*) and goat grass (*Aegilops tauschii* Coss), leading to so‐called synthetic hexaploid wheat (SHW). We applied the quantitative genetics tools of “hybrid prediction”—originally developed for the prediction of wheat hybrids generated from different heterotic groups — to a situation of allopolyploidization. Our use‐case predicts the phenotypes of SHW for three quantitatively inherited global wheat diseases, namely tan spot (TS), septoria nodorum blotch (SNB), and spot blotch (SB). Our results revealed prediction abilities comparable to studies in ‘traditional’ elite or hybrid wheat. Prediction abilities were highest using a marker model and performing random cross‐validation, predicting the performance of untested SHW (0.483 for SB to 0.730 for TS). When testing parents not necessarily used in SHW, combination prediction abilities were slightly lower (0.378 for SB to 0.718 for TS), yet still promising. Despite the limited phenotypic data, our results provide a general example for predictive models targeting an allopolyploidization event and a method that can guide the use of genetic resources available in gene banks.

## Core Ideas

- Genomic prediction of synthetic hexaploid wheat (SHW) from tetraploid durum wheat and diploid *Aegilops* parental pools was investigated.
- To widen the genetic base in breeding, bread wheat has been re‐synthesized by crossing durum wheat and goat grass.
- We applied the “hybrid prediction” to a situation of allopolyploidization.
- Our use‐case predicts the phenotypes of SHW for three quantitatively inherited wheat diseases.

**Abbreviations**
CIMMYT: International Maize and Wheat Improvement Center
CV1: cross‐validation 1
CV2: cross‐validation 2
CV3: cross‐validation 3
DW: durum wheat
GBLUP: genomic best linear unbiased predictor
GCA: general combining ability
GK: Gaussian kernel
GM: genomic model
PGM‐1: pedigree + genomic model
PGM‐2: pedigree + genomic model
PGM‐3: pedigree + genomic model
PM: pedigree model
SB: spot blotch
SCA: specific combining ability
SHW: synthetic hexaploid wheat
SNB: septoria nodorum blotch
SNP: single nucleotide polimorphism
TS: tan spot

## 1 INTRODUCTION

Polyploidy, a prevalent occurrence in the Plantae kingdom, refers to the condition in which a cell has more than two copies of each homologous chromosome. While the evolutionary advantage of polyploidy remains a topic of discussion, it is commonly associated with amplified growth vigor and is believed to enhance the adaptive potential of species (Van de Peer et al., 2021, 2017). Several major food crops are polyploids, either as allopolyploids such as cotton, rapeseed, and wheat or as autopolyploids, as seen in the majority of commercial potato varieties (Chen, 2007). Allopolyploidy refers to the merging of the genomes of two (or more) different species, and it involves intergenomic interaction, which may produce new phenotypic variation and heterosis (Chen, 2007; Ni et al., 2009).

Bread wheat (*Triticum aestivum* L.) is an allohexaploid that was formed between 8000 and 10,000 years ago as a hybrid between the early cultivated allotetraploid *Triticum turgidum* ssp. *durum* Desf. MacKey (2*n* = 4*x* = 28, AABB) and the diploid goat grass *Aegilops tauschii* Coss (2*n* = 2*x* = 14, DD), followed by spontaneous chromosome doubling (Kihara, 1944). After its domestication, hexaploid wheat emerged as a superior form, surpassing its tetraploid and diploid ancestors. Hexaploid wheat grows more robustly, confers broader adaptability, provides an enhanced ability against pathogens, and offers versatile end products (Dubcovsky & Dvorak, 2007). Through centuries of cultivation and selection, hexaploid wheat has become a staple food crop.

Global wheat production amounts to ∼770 metric tons annually (FAO, 2020. Over two‐thirds of its production is devoted to food consumption. This significant contribution establishes wheat as a vital dietary component for an estimated 35% of the global population (Grote et al., 2021; Shiferaw et al., 2013). Considering the expected increase in the demand for wheat—to keep up with the world's increasing population and to counter global crises and unrest that trigger volatile markets—accelerated wheat improvement and enhanced productivity are required. Moreover, global warming has already become a worldwide threat, with more frequent and extreme weather events, including severe droughts or heat stress (Gaupp et al., 2020; Kornhuber et al., 2020; Sarhadi et al., 2018). New pathogen strains are emerging more frequently, posing difficult challenges for breeding new advanced wheat varieties and global wheat cultivation (Nnadi & Carter, 2021).

**FIGURE 1:**
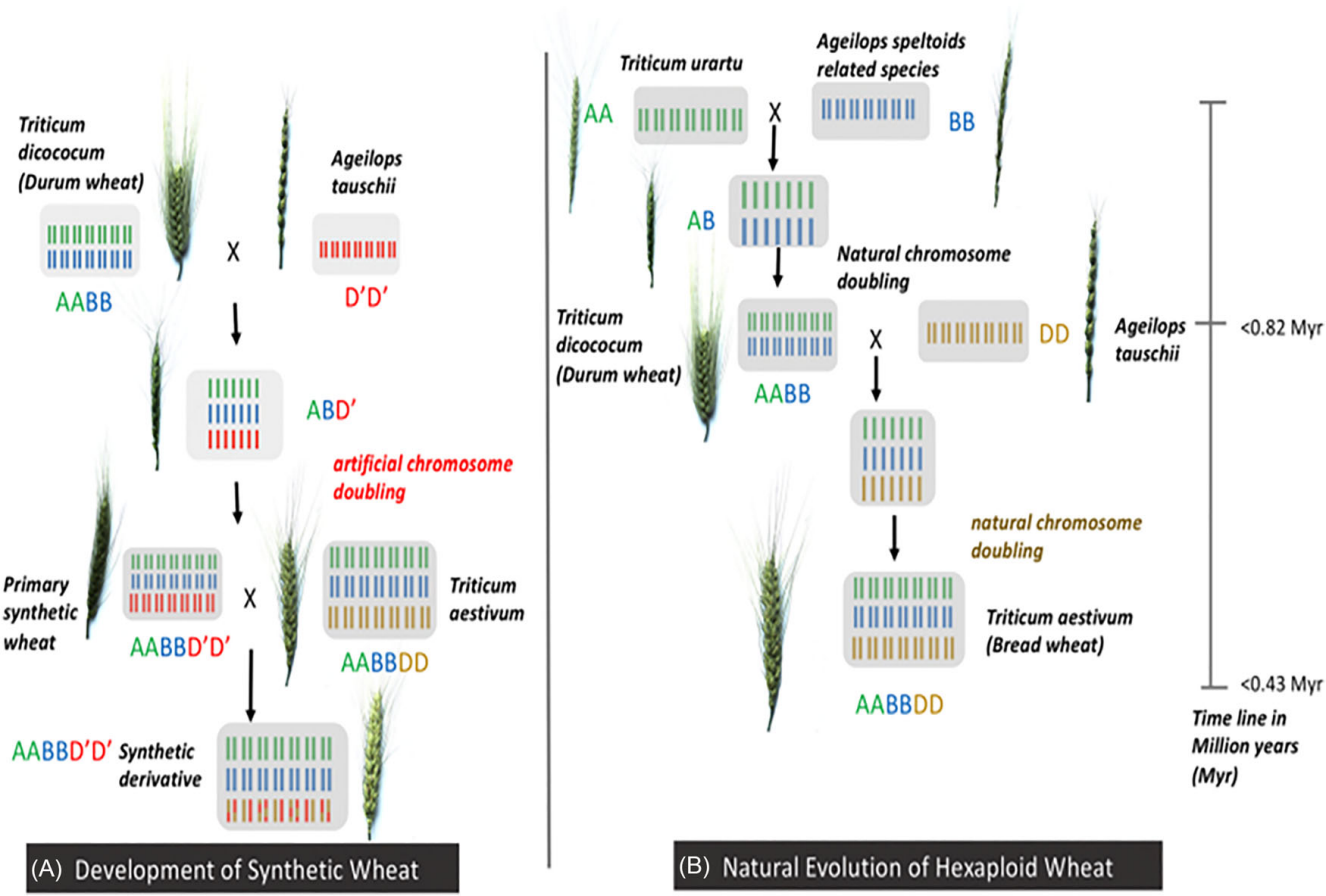
Development of synthetic hexaploidy wheat in comparison with the natural evolution of bread wheat (adopted from Rosyara et al., 2019).

The polyploidization event that bread wheat underwent in its domestication history can be repeated (“synthesized”) by interspecific hybridization followed by spontaneous chromosome doubling (Mujeeb‐Kazi & Hettel, 1995; Figure 1). This artificially formed nascent bread wheat is named “synthetic hexaploid wheat” (SHW). SHW can be designed to capture novel genetic diversity from tetraploid and diploid relatives of wheat that is not found in “traditional” bread wheat. With no significant reproduction barrier, SHW lines are useful to introduce agronomically needed traits into bread wheat from available genetic resources (Singh et al., 2018).

SHW often exhibits increased resilience to biotic stresses and thus provides an opportunity to discover a new resistance to many pathogens (Aberkane et al., 2020). In particular, wheat foliar diseases have become increasingly importance in recent years due to various factors, such as the threat of climate change and the widespread cultivation of susceptible wheat varieties on a commercial scale (Lozano‐Ramirez et al., 2022a; Singh et al., 2010). The causal agents of the diseases infect multiple wheat tissues, such as root, stem, leaf, spike, and grain. Based on the frequency and severity levels of disease epidemics, the diseases that infect leaf and spike are considered more important (Lozano‐Ramirez et al., 2022a). Some of the most important foliar diseases are “tan spot” (TS), caused by *Pyrenophora tritici‐repentis* Drechs (anamorph *Drechslera tritici‐repentis* Shoem.); “septoria nodorum blotch” (SNB), caused by *Parastagonospora nodorum* (syn. ana, *Stagonospora*; teleo. Phaeosphaeria) (Berk.) Quaedvlieg, Verkley, and Crous; and “spot blotch” (SB), caused by *Bipolaris sorokiniana*.

While many SHWs are characterized by the resistance to diverse pathogens, it has been difficult to forecast the resistance phenotype based on the relative performance of the parents. Possible contributing factors reported in previous studies are distinct levels of genetic and epigenetic alternations, changing gene expression patterns, and aneuploidy during the synthesis of SHW (Chen et al., 2023; Eagles et al., 2011; Li et al., 2018; Mestiri et al., 2010; Vasudevan et al., 2023; Yu et al., 2017). In addition, the nature and growth of the wild *Ae. tauschii* species inhibit reliable phenotypic screening, which means that the degree of resistance/susceptibility of the relevant parent itself cannot be characterized. The main criteria to select *Ae. tauschii* as a parent to synthesize a novel SHW have therefore been focused on capturing diverse material from accessions in gene bank collections, facing the key challenge of countless cross‐combination options (Aberkane et al., 2020).

The prediction of the performance of hybrid combinations of different heterotic groups, such as the one used in maize (Technow et al., 2012), involves a similar task of predicting the most promising combinations of parents, only within and not between species. Genomics‐enabled prediction, including pedigree and marker information, has recently shown reliable results in the prediction of hybrid performance from the wheat parents forming the single crosses (Basnet et al., 2019; Rembe et al., 2019; Zhao et al., 2013). Genomic‐enabled prediction methods could also help harness the genetic diversity provided by tetraploid wheat and *Ae. tauschii* collections as a special case of the general attempt to use predictive breeding methods to harness the potential of germplasm collections (Martini et al., 2021; Yu et al., 2016). In this research, we aimed to model the variation of SHW quantitatively, with genomic hybrid prediction considering durum wheat (DW) and *Ae. tauschii* parents as two different parental pools.

**Core Ideas**

- Genomic prediction of synthetic hexaploid wheat (SHW) from tetraploid durum wheat and diploid *Aegilops* parental pools was investigated.
- To widen the genetic base in breeding, bread wheat has been re‐synthesized by crossing durum wheat and goat grass.
- We applied the “hybrid prediction” to a situation of allopolyploidization.
- Our use‐case predicts the phenotypes of SHW for three quantitatively inherited wheat diseases.

## 2 MATERIALS AND METHODS

### 2.1 Plant materials inoculation procedures and phenotypic data measurements

A total of 443 SHW lines were available (Table S1), derived from an interspecific cross between DW (2*n* = 4*x* = 28, AABB, *T. turgidum* L.) and goat grass (2*n* = 2*x* = 14, DD, *A. tauschii* Coss.) (Figure 1). The lines were created by the International Maize and Wheat Improvement Center (CIMMYT) in its “Wheat Wide Crosses Program” between 1988 and 2010. The SHW included 57 different DW parents and 281 *Ae. tauschii* accessions (Table S1).

The SHW, along with the DW parents, was evaluated for four globally important diseases: (1) TS, caused by *P. tritici‐repentis*, a foliar disease that has become a major threat within the complex of leaf spotting diseases and has increased worldwide over the last several decades; (2) SNB, caused by *P. nodorum*, which occurs in wheat‐growing areas worldwide, and especially in areas with warm and moist weather; and (3) SB, from the fungal pathogen *B. sorokiniana*, that causes enormous losses in areas with climates characterized by high temperatures and humidity at late growth stages such as in eastern India, Southeast Asia, Latin America, Nepal, China, and Africa.

The assessment of the leaf‐spotting diseases (TS, SNB, and SB) was performed under greenhouse conditions at the seedling stage in the CIMMYT headquarters in El Batan, Mexico (19°31′N, 98°50′W; elevation of 2249 m above sea level) in 2018–2019. The SHW and DW seeds were scarified to break dormancy and obtain an even germination. The experiments were arranged in a randomized complete block design with 12 replicates for TS and SNB and six replicates for SB. For each entry, four plants were grown in plastic containers as experimental units to derive mean values for subsequent analysis. The seedlings were grown under controlled environmental conditions with an air temperature of 22–25°C/16–18°C (day/night) and a 16‐h photoperiod. Checks for TS and SNB evaluation included the bread wheat cultivars Erik (resistant), 6B‐662 (moderately resistant), 6B‐365 (moderately susceptible), and Glenlea (susceptible). For SB, checks used were Chirya (resistant), Francolin (moderately susceptible), Ciano F79 (susceptible), and Sonalika (susceptible).

For TS, the Mexican PTR isolate CIMFU 531‐PTR1 (race 1) was applied. The isolate was grown in a V8‐PDA medium (Lamari & Bernier, 1989), and conidia concentration for inoculation was adjusted to 4 × 10^3^ spores mL^−1^ using a Fuchs‐Rosenthal counting chamber. One drop of Tween 20 (a surfactant reagent) was added to every 100 mL of the spore suspension. The Mexican isolate CIMFU463‐SN4 was used to evaluate SNB resistance. The isolate was adjusted to a concentration of 10^7^ conidia per milliliter in a Neubauer counting chamber. One milliliter of Tween 20 was added to every 100 mL of isolate suspension for inoculation. Similarly, the Mexican isolate CIMFU483‐BSG4M2 was used for the inoculation of SB. This isolate was adjusted to a concentration of 7500 conidia per milliliter in the same Neubauer chamber, and 1 mL Tween 20 was added to every 100 mL isolate suspension for inoculation.

The seedlings were inoculated with the respective conidial suspension for every disease in the two‐leaf stage when the second leaf was fully expanded, or 2 weeks after sowing. Isolates were induced until the leaves were at a dew point, and this process was carried out four times using a hand sprayer. When the leaves were dry, the trays were moved to a mist chamber (relative humidity 100%, 21–22°C) to facilitate infection. After 24 h (48 h in the case of SB), the plants were transferred back to the greenhouse bench. Seedling response was evaluated 7 days after inoculation by following the 1–5 lesion rating scale developed by Lamari and Bernier (1989), which is based on the lesion type shown on the secondary leaf where 1 indicates resistant, 2 indicates moderately resistant, 3 indicates moderately resistant to moderately susceptible, 4 indicates susceptible, and 5 indicates highly susceptible.

### 2.2 DNA isolation and sequencing

Fresh young leaves bulked from five individual plants per SHW were used for DNA extraction using a modified cetyltrimethylammonium bromide method (Dreisigacker et al., 2016). The DNA was quantified with a NanoDrop 8000 spectrophotometer V 2.1.0, and all entries were genotyped with the DArTseq technology at the Genetic Analysis Service for Agriculture (SAGA) laboratory in CIMMYT in Mexico (Sansaloni et al., 2011). For this study, we excluded single nucleotide polymorphisms (SNPs) with >15% of missing values and minor allele frequencies of 5%; missing genotypes were imputed with samples from the marginal distribution of observed marker genotypes based on the sample mean of each marker already recoded to its corresponding numeric values (0,1,2). The final 4169 high‐quality SNPs were used for further analyses. Seeds from the DW and *Ae. tauschii* parents were, in many cases, no longer accessible. Searching for historical genotypic data generated by the CIMMYT gene bank revealed SNP data for only a limited number of 32 DW parents and 27 *Ae. tauschii* accessions, which formed a total of 147 SHW. SNP data of parental lines were therefore not used.

### 2.3 Statistical models

Two stages were used in this study. In the first stage, a phenotypic ordinal linear model was fitted with the aim of estimating the effects of the SHW cultivars ($l_{i})$ (Alvarado et al., 2020). In the second stage, models for the genomic adjustment and prediction of missing cultivars were applied. Both phenotypic and genomic models presented were fitted using the BGLR package (Pérez & de los Campos, 2014), comprising 50,000 iterations. The initial 5000 iterations were discarded, and a thinning of 5 was applied to minimize random errors.

### 2.4 Phenotypic model for disease traits TS, SNB, and SB

We estimated the effects of the SHW cultivars ($l_{i}$), using a linear model for ordinal traits, where the response can take values on C ordered values, $y_{ij}\in \left\{1,\text{…},C\right\}$. We used the probit link, and the probability of each observation belonging to each category is given by:

(1) $$P\;\left(\;y_{ij}=c\right)=\;\Phi \left(\eta_{ij}-\gamma_{c}\right)-\Phi \left(\eta_{ij}-\gamma_{c-1}\right),$$

where Φ(·) is the cumulative distribution function of a standard normal random variable, $\eta_{ij}=r_{j}+l_{i}$, which corresponds to the linear predictor, which includes the effect of replicates ($r_{j})$ and SHW lines $(l_{i}$); $\gamma_{c}$ are threshold parameters, with $\gamma_{0}=-\infty ,\;\gamma_{c}\geq \gamma_{c-1},\;\;\gamma_{C}=\infty$. The model was fitted using the BGLR package (Pérez & de los Campos, 2014) using data augmentation; that is, the latent variables (liabilities) $L_{ij}$ are introduced in the model as follows: $L_{ij}=\eta_{ij}+\varepsilon_{ij}$, where $\varepsilon_{ij}$ are independent and identically distributed random variables with mean 0 and variance 1. For more details about the threshold model, see Gianola (1982).

**FIGURE 2:**
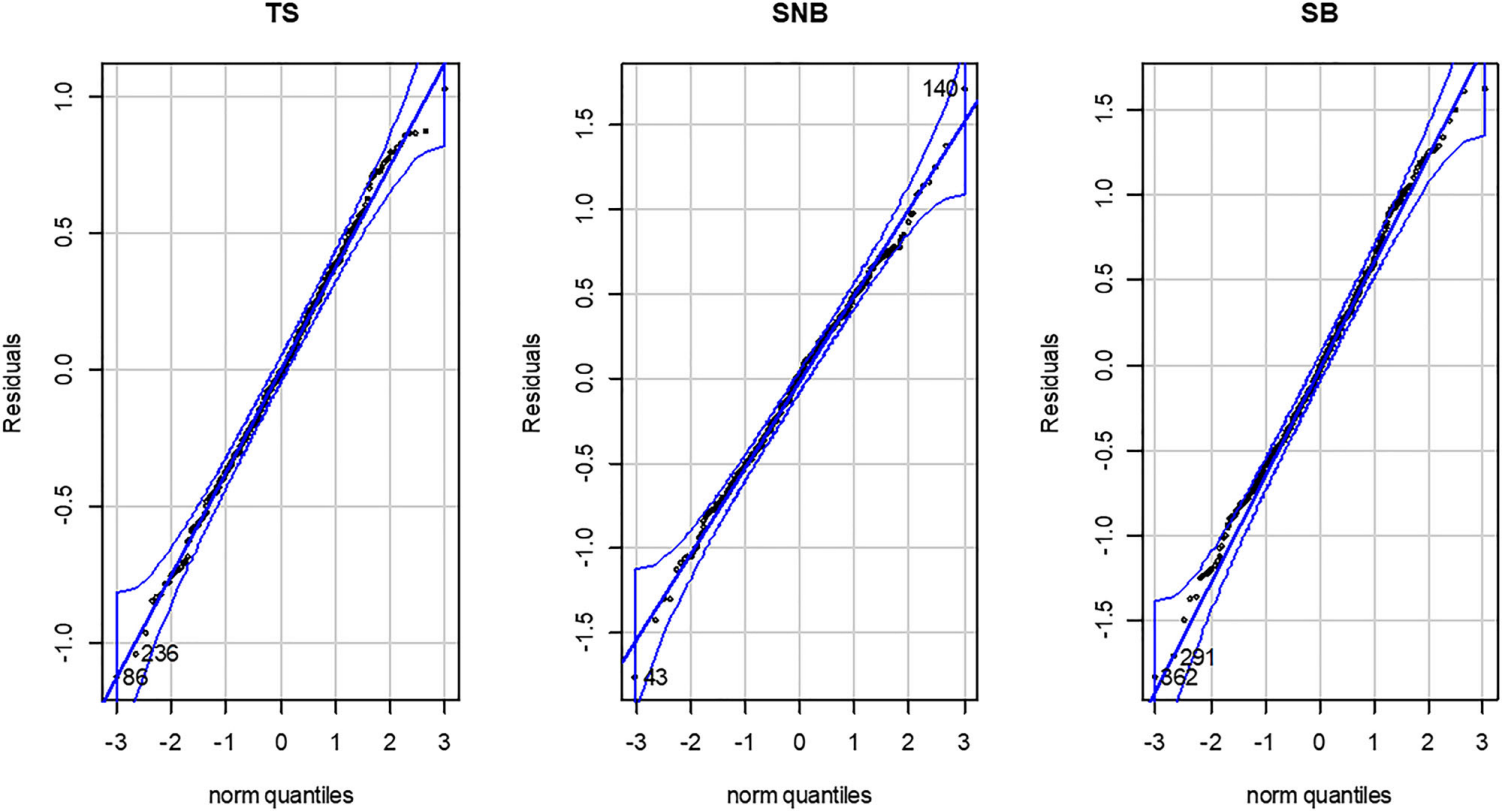
Residuals with a normal distribution obtained when fitting genomic model (GM) (model 2) for traits tan spot (TS), septoria nodorum blotch (SNB), and spot blotch (SB).

Note that the effects of cultivars ($l_{i}$) computed in stage 1 are considered response variables to be used in stage 2 when performing the genomic prediction. In stage 2 (see the description of the various genomic models below), the response for traits TS, SNB, and SB obtained from stage 1 is considered a continuous variable with a symmetric distribution that guarantees a normal distribution of the residuals (Figure 2).

### 2.5 Genomic prediction models with pedigree and markers

Pedigree information was available for the DW parents and the SHW lines. The accession names of the *Ae. tauschii* parents were adopted assuming no pedigree relationship. We created prediction models that included normally distributed random terms with covariance defined by pedigree or genomic information. Thus, models implemented in this study attempt to capture (1) additive gene effects from the two parents, DW and *Ae. tauschii* (general combining ability, GCA), and (2) nonadditive effects due to the DW × *Ae. tauschii* cross to represent variability due to intra‐locus interaction (dominance) and inter‐loci interaction (epistasis) (specific combining ability, SCA).

The metric used to assess the model's prediction ability is the average Pearson's correlation between observed and predicted values. In this study, a fivefold random cross‐validation (CV) was used to obtain the Pearson's correlation. Genomic prediction was assessed considering three main random CVs explained below. Model comparisons were carried out for the mean differences between the various pedigree model (PM), genomic model (GM), PGM‐1, PGM‐2, and PGM‐3 models.

#### 2.5.1 Pedigree model (PM)

Only the pedigree information represented by the numerical relationship matrix (***A***) was used in this model to determine the genetic additive relationship between DW parents, *Ae. tauschii* parents, and their crosses forming the SHW. The relationship matrix was derived by the coefficient of co‐ancestry computed using the BROWSE software (McLaren et al., 2005, 2000). The full genetic model to assess the GCA of the DW and *Ae. tauschii* parents and their SCA is described by the following linear mixed model:

(2) $$y=\mu 1+Z_{d_{p}}d_{p}+Z_{t_{I}}t_{I}+Z_{c_{p}}c_{p}+e,$$

where matrices $Z_{d_{p}},\;Z_{t_{I}},\;\mathrm{and}\;Z_{c_{p}}$ are the incident matrices that relate the values of random vectors $d_{p},t_{I},\;\mathrm{and}\;c_{p}$ to the response variable y, μ is an intercept (fixed effect), $d_{p}∼\mathrm{MN}\left(0,\sigma_{d_{p}}^{2}A_{d}\right)$ is the random vector for the GCA of the DW (*d* subscript) obtained from the numerator relationship matrix ($A_{d}$) derived from the coefficient of coancestry (the subscript *p* denotes pedigree) and assumed to be distributed following a multivariate normal (MN) random variable with $\sigma_{d_{p}}^{2}$ as the additive parameter variance estimated from the PM. The term $t_{I}∼\mathrm{MN}\left(0,\sigma_{t_{I}}^{2}I\right)$ is a random vector for the GCA of the *Ae. Tauschii* parents (*t* subscript) assuming that they are normal, independent, and identically distributed (*niid*) with a mean of 0 and a variance of $\sigma_{t_{I}}^{2}$ being the additive variance for the *Ae. tauschii* parents with no relationship (***I*** denotes the identity matrix). Furthermore, the term $c_{p}∼\mathrm{MN}\left(0,\sigma_{c_{p}}^{2}A_{c}\right)$ is the random vector of the SCA of the DW × *Ae. tauschii* crosses forming SHW. The relationship matrix $(A_{c}$) is derived from the coefficient of co‐ancestry (subscript *p* denotes pedigree and subscript *c*, the cross). The variance component associated with the cross is identified as $\sigma_{c_{p}}^{2}$. The random residual term e is distributed as $\mathrm{MN}(0,\sigma_{e}^{2}I)$ with $\sigma_{e}^{2}$ being the variance of the residuals. We also assume that the random terms $d_{p},\;t_{I},\;c_{p},$ and e are independent.

#### 2.5.2 Genomic model (GM)

The information in this model only uses the SNP data of the SHW lines because only limited SNP data for parental lines were available. The linear model is given by:

(3) $$y=\mu 1+Z_{c_{m}}c_{m}+e,$$

where $c_{m}∼\mathrm{MN}\left(0,\sigma_{c_{m}}^{2}K_{c}\right)$ is the random vector of the SHWs, and $Z_{c_{m}}$ is the incidence matrix that relates the random vector $c_{m}$ with the phenotype data. The genomic relationship matrix $(K_{c}$) is derived from the DArTSeq markers (subscript *m* denotes markers and subscript *c*, the cross). $\sigma_{c_{m}}^{2}$ is the variance of the cross. Let X be a c×p matrix of standardized markers (mean zero and variance one for each marker), where c is the number of crosses (individuals) and p is the number of markers.

We defined two kernel matrices of genomic relationship: (1) the first kernel matrix is linear $K={\mathrm{XX}}^{\prime }/p$ (Lopez‐Cruz et al., 2015), and it is the relationship matrix used in the genomic best linear unbiased predictor (GBLUP) (GB) model and (2) the second kernel matrix is nonlinear and is constructed with each element computed as $K_{ii^{\prime }}=\exp(-h\times \mathrm{dist}\left(x_{i},x_{i^{\prime }}\right)$ where $\mathrm{dist}(x_{i},x_{i^{\prime }})$ is the squared distance between two rows $x_{i},x_{i^{\prime }}$, $i,\;i^{\prime }=1,\text{…},n$ (number of records with genotypic information) and h is two times the reciprocal of the median of the squared distances; this nonlinear kernel is named Gaussian kernel (GK) (Gianola & van Haam, 2008) or reproducing kernel Hilbert spaces (Crossa et al., 2010).

#### 2.5.3 Pedigree + genomic model (PGM‐1)

By combining PM (Equation 2) and GM (Equation 3), we obtain model PGM‐1

(4) $$y=\mu 1+Z_{d_{p}}d_{p}+Z_{t_{I}}t_{I}+Z_{c_{p}}c_{p}+Z_{c_{m}}c_{m}+e,$$

where all the components are already defined above.

#### 2.5.4 Pedigree + genomic model (PGM‐2) (single‐step approach developing the ***H*** matrix)

This model considers the information of the markers and pedigree using the single step approach by Legarra et al. (2009) and Aguilar et al. (2010). The PGM‐2 model is given by:

(5) $$y=\mu 1+Z_{h_{d}}h_{d}+Z_{h_{t}}h_{t}+Z_{h_{c}}h_{c}+e,$$

where $Z_{h_{d}},\;Z_{h_{t}},\;\mathrm{and}\;Z_{h_{c}}$ are incidence matrices that relate observations with the random vectors $h_{d},\;h_{t},\;\mathrm{and}\;h_{c}$, respectively; $h_{d}∼\mathrm{MN}\left(0,\sigma_{h_{d}}^{2}H_{d}\right)$ is a random vector for the general combining ability of the DW (*d*) parents, where $\sigma_{H_{d}}^{2}$ is the variance of the DW parents that is the parameter associated with the matrix $H_{d}$ that includes pedigree and genomic information for the DW lines; $h_{t}∼\mathrm{MN}\left(0,\sigma_{h_{t}}^{2}H_{t}\right)$ is a random vector for the general combining ability of the *Ae. tauschii* (*t*) parents, where $\sigma_{H_{t}}^{2}$ is the variance of the *Ae. tauschii* parents that is the parameter associated with the matrix $H_{t}$ for *Ae. tauschii* lines; $h_{c}∼\mathrm{MN}\left(0,\sigma_{h_{c}}^{2}H_{c}\right)$ is the random vector of specific combining ability of the DW × *Ae. tauschii* crosses (*c*), where $\sigma_{c_{H}}^{2}$ is the variance of the cross (DW × *Ae. tauschii*) that is the parameter attached to the joined pedigree and genomic similarity matrix $H_{c}$ for the cross.

Matrices ***H*** were generated using the relationship matrix derived from pedigree (***A***) and a relationship matrix derived from markers (***K***) according to Legarra et al. (2009) and Aguilar et al. (2010), and is given by:

(6) $$H=\left[\begin{array}{cc} H_{11} & H_{12}\\ H_{21} & H_{22} \end{array}\right]\;,$$

where $H_{11}=A_{11}\;+A_{12}A_{22}^{-1}(K-A_{22}$)$\;A_{22}^{-1}A_{21},\;\;H_{12}=A_{12}\;A_{22}^{-1}K$, $H_{22}=K$, $H_{21}=H_{12}^{\prime }$. The matrix is divided according to whether the individuals are genotyped or not; 1 denotes the individuals that were not genotyped, and 2 denotes the individuals genotyped, so that, for example, $A_{21}$ represents the relationship between genotyped and non‐genotyped individuals (Pérez‐Rodríguez et al., 2017).

According to Christensen (2012), matrix K should be adjusted in order to have the same scale as matrix ***A***, that is:

(7) $$\;K_{a}=\beta K+\alpha ,$$

where β and α are obtained by solving the following system of equations:

(8) $$\mathrm{Avg}\left(\mathrm{diag}\left(K\right)\right)\beta +\alpha =\;\mathrm{Avg}\left(\mathrm{diag}\left(A_{22}\right)\right),$$

(9) $$\mathrm{Avg}\left(K\right)\beta +\alpha =\mathrm{Avg}\left(A_{22}\right).$$

#### 2.5.5 Pedigree + genomic model (PGM‐3) (interactions DW × *Ae. tauschii*)

This PGM‐3 model considers the information of pedigree and markers based on the single step of Legarra et al. (2009) and Aguilar et al. (2010) in combination with the perspective provided by Acosta‐Pech et al. (2017) for the hybrid prediction from the interaction combination of the parents.

(10) $$y=\mu 1+h_{\mathrm{dxt}}+Z_{h_{c}}h_{c}+e,$$

where 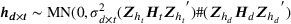 is the random vector of interaction between the parents from DW and *Ae. tauschii*, $\sigma_{d\times t}^{2}$ is the variance component and matrices $Z_{h_{t}},\;Z_{h_{d}},H_{t},H_{d}$ were defined earlier, with # being the Hadamard product. Acosta et al. (2017) suggested a Kronecker product, but since the data of this study are not balanced, the equivalent operation is the Hadamard product (Martini et al., 2020); $h_{c}∼\mathrm{MN}\left(0,\sigma_{h_{c}}^{2}H_{c}\right)$ is the random vector of SCA of the DW × *Ae. tauschii* crosses, where $\sigma_{h_{c}}^{2}$ is the variance of the cross (DW × *Ae. tauschii*) that is the scaled parameter associated with the joint pedigree and genomic similarity matrix $H_{c}$ for the cross.

### 2.6 Cross‐validation schemes

**FIGURE 3:**
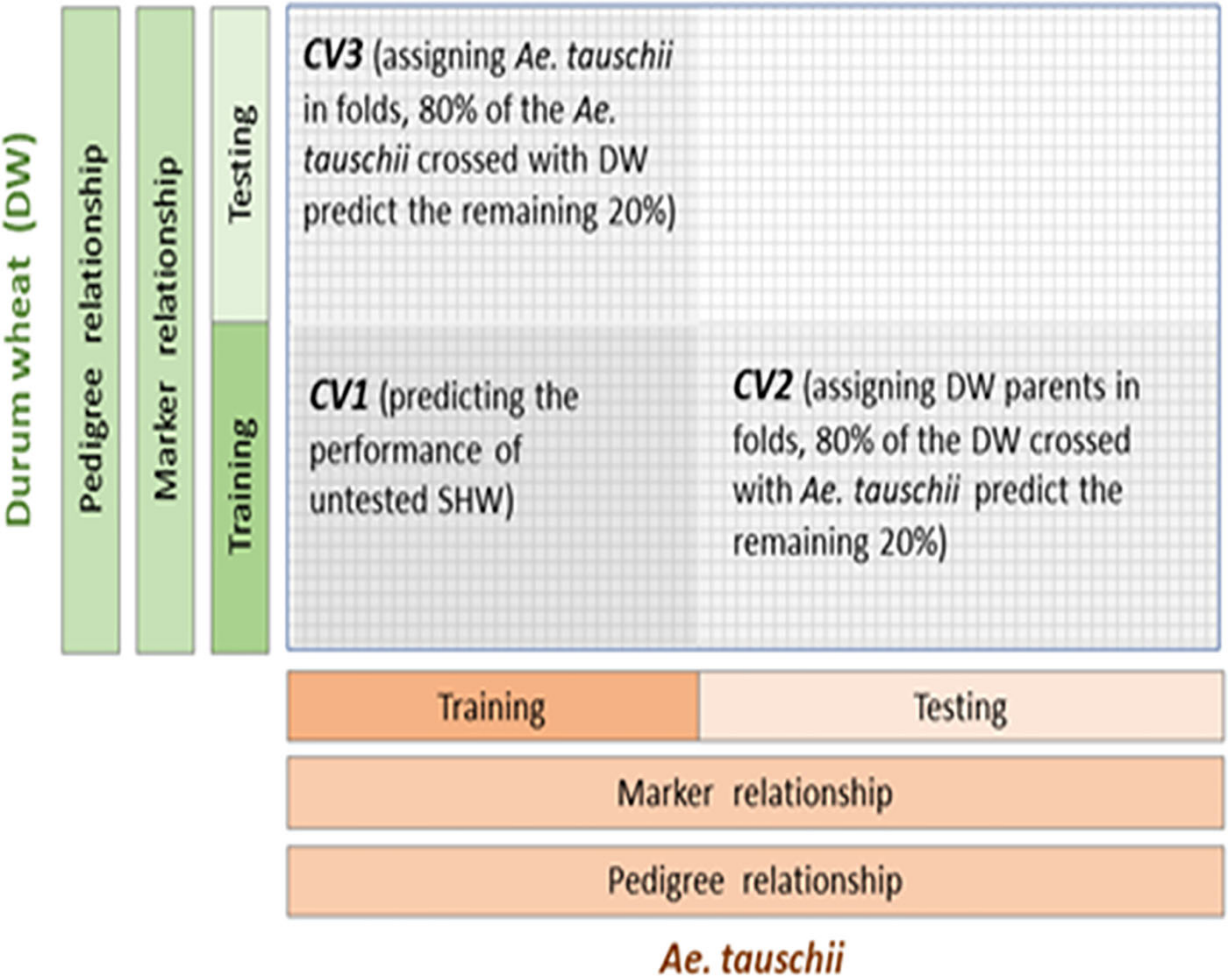
Three prediction problems of tested and untested synthetic hexaploid wheat (SHW) and their parents. CV1 tested SHW (DW × *Ae. tauschii*) using fivefold random cross‐validation with 80% of data in the training set and 20% in the testing set. CV2 leave 80% of the DW parents out and predict the SHW with the remaining 20% DW parents. CV3 leave 80% of the *Ae. tauschii* parents out and predict the SHW with the remaining 20% *Ae. tauschii* parents. DW, durum wheat.

We studied several genomic prediction problems related to approaches breeders or scientists might deploy when evaluating or generating new SHW. Three types of prediction problems were assessed, similar or related to prediction problems used in hybrid breeding, as in Basnet et al. (2019), as shown in Figure 3. The three CVs include different prediction situations.

#### 2.6.1 CV1: Assign SHW to five folds

CV1 studies the prediction of the SHW when both parents, DW and *Ae. tauschii*, are known. We performed a random CV analysis using a scheme that is known as cross‐validation CV1 (Burgueño et al., 2012), which considers the prediction of a certain proportion of untested cultivars. In our study, we assume several SHW are assessed for disease resistance, while for other SHWs, phenotypic values are unobserved (missing). Overall, more than 1000 SHW have been generated at the CIMMYT since the 1980s, but only a subset of SHW has been evaluated for individual traits; thus, CV1 reflects the problem breeders face of usually not having the full capacity to evaluate all possible cultivars (germplasm) for all type of target traits.

#### 2.6.2 CV2: Assign DW parent to five folds

CV2 investigates the prediction of SHW when one parent, *Ae. tauschii*, is known, but a proportion (20%) of the DW parent is unobserved. We performed CV assigning the DW parents to five folds; therefore, if a set of DW lines is assigned to the training (testing) set, then all the corresponding SHW lines were assigned to the training (testing) set. The training set includes all the SHW lines obtained when using 80% of the DW and predicting the remaining 20% of SHW (from unobserved DW). This CV scheme mimics the problem of predicting SHW whose DW parent has not yet been observed in any SHW combination. Since the number of SHW was not equal across DW, the SHW lines in training and testing sets vary.

#### 2.6.3 CV3: Assign *Ae. tauschii* parents to five folds

CV3 comprises the prediction of SHW when one parent, DW, is known, but a proportion (20%) of the parent *Ae. tauschii* is unobserved. Similar to CV2, we performed CV assigning the *Ae. tauschii* wheat parents to folds. In general, a larger number of *Ae. tauschii* parents are used when generating new SHW. Here, the training set includes all the SHW obtained when using 80% of the *Ae. tauschii* crossed with durum wheat and predicting the remaining 20% of the SHW. This CV scheme reflects the problem of predicting SHW using DW parents whose crosses with any of the *Ae. tauschii* accessions were not yet observed.

### 2.7 Heritability based on genomic model

To compute heritability based on genomics, we considered the proposal by Feldman et al. (2022) that attempts to improve the precision of a heritability estimate by using the concept of average semi‐variance. It is assumed that the SHW cultivars represent a random effect with identical and independent distributions (*iid*) $g_{i}\overset{iid}{∼}N\left(0,\;K_{\mathrm{ASV}}\sigma_{g}^{2}\right)$, where $K_{\mathrm{ASV}}$ is the relationship matrix computed following Feldman et al. (2022); the variance of the SHW is represented as $\sigma_{g}^{2}$. Then, the heritability is

(11) $$\;h^{2}=\frac{\sigma_{g}^{2}}{\sigma_{g}^{2}+\sigma_{\varepsilon }^{2}},$$

where the variance component $\sigma_{\varepsilon }^{2}$ denotes the residual error variance when assuming residuals being normally independent and identically distributed.

## 3 RESULTS AND DISCUSSION

Data analyses were based on the phenotypic effects of the SHW across the 12 replicates for disease traits TS, SNB, and the six replicates for the disease trait SB. Note that the replicates were established in the greenhouse and sown at different planting dates.

For each of the models described above, the two kernels (GB and GK) and three types of CVs (CV1, CV2, and CV3) were used. For the SHW and DW parents, the pedigree information was available, while the *Ae. tauschii* accessions were assumed to be unrelated. SNP data for all SHW were generated in the study by Lozano‐Ramirez et al. (2022a). Table 1 shows that the SNP data on the parental lines were only available for 32 DW parents and 127 *Ae. tauschii* accessions forming a total of 147 out of the 386 SHW used. The limited data availability for the parental lines justifies the use of the single step models (PGM‐2 and PGM‐3).

**TABLE 1:** Information from adjusted means, genomic heritability, and sample size for each trait: tan spot (TS), septoria nodorum blotch (SNB), and spot blotch (SB).

				Available sample size					
	Adjusted means			SHW		DW		*Ae. tauschii*	
Trait	Mean	SD	Heritability	Pedigree	Markers	Pedigree	Markers	Identity	Markers
TS	1.53	0.68	0.64	386	386	39	32	264	127
SNB	1.70	0.71	0.67	386	386	39	32	264	127
SB	1.51	0.58	0.57	386	386	39	32	264	127

*Note*: Details of the inoculation procedures for each disease are given in Section 2.

Abbreviations: DW, durum wheat; SHW, synthetic hexaploid wheat.

For genomic adjustment and prediction, two stages were carried out. In the first stage, the adjusted means of the cultivars were estimated using an ordinal response model. Table 1 displays the results of this adjustment, showing the means and standard deviations. Additionally, skewness coefficients of the adjusted means were calculated, resulting in 0.20, 0.15, and 0.25 for TS, SNB, and SB, respectively, indicating only small asymmetries. Similarly, variances of the adjusted means were calculated, with mean variances of 0.11, 0.12, and 0.16, as well as standard deviations of 0.03, 0.05, and 0.05 for TS, SNB, and SB, respectively. Based on the results, it can be assumed that the adjusted means follow a symmetric distribution with homogeneous variances.

Additionally, the homogeneity of residual variances was tested using the proposal by Breusch and Pagan (1979). In a simplified form, this involves fitting a regression model with the squared residuals from the original model as the response variable and the covariates from the original model as predictors. With this adjusted model, a statistic is calculated, equal to the number of observations (*n*) multiplied by the coefficient of determination. Subsequently, a *p*‐value is determined using the *χ* ^2^ distribution with degrees of freedom equal to the number of covariates in the model. In all cases, the *p*‐value of this test was close to 1, indicating no evidence to reject the null hypothesis of a constant variance existing in the residuals.

We reiterate that for stage 2, genomic prediction models are fitted for traits TS, SNB, and SB assuming the responses are continuous, and data are from a normal distribution such that the residuals of the models have a normal distribution as shown in Figure 2, which shows that all the residuals are contained in the corresponding confidence intervals. Furthermore, for the three diseases (TS, SNB, and SB), the Shapiro test for normality indicated evidence of not rejecting the null hypothesis that the residuals come from normal distribution.

### 3.1 Resistance scores and variance components

The 386 SHW evaluated in the greenhouse displayed high levels of resistance and moderate resistance for all four foliar diseases (Table 1), and reaction types were comparable to the disease‐specific resistance checks (data not shown). The means for the TS, SNB, and SB disease scores were similarly high. Heritability estimates for the traits were similar except for trait SB with a heritability of 0.53. The DW parents that were evaluated for the same diseases in parallel showed overall lower levels of resistance (data not shown), while the *Ae. tauschii* parents could not be screened. The lower resistance scores in the DW parents suggest that the resistance in the SHW lines was derived from both the DW and *Ae. tauschii* parents. Genome‐wide association studies conducted on the same data sets confirmed this result and showed that genomic loci from all three genomes played a role in conferring resistance within the SHW lines (Lozano‐Ramirez et al., 2022a; Lozano‐Ramirez et al., 2022b). Higher levels of Fusarium crown rot resistance in SHW compared with their tetraploid parents were also observed by Chen et al. (2023). The authors validated that phenylalanine ammonia lyase genes, involved in the biosynthesis of lignin and salicylic acid, displayed a higher level of expression to Fusarium crown rot infection in the SHWs, leading to improved resistance.

Variance components of the fitted models (PM, GM, PGM‐1, PGM‐2, and PGM‐3) for all three diseases were estimated (Table 2). The characterization of the variance components due to GCA and SCA is of central importance in hybrid prediction. GCA is directly related to the breeding value of a parent and is associated with additive genetic effects, while SCA is the relative performance of cross that is associated with nonadditive gene action, predominantly contributed by dominance and epistasis, suggesting the use of a nonlinear GK that enables accounting for complex interaction (Crossa et al., 2010). The variance explained by the sub‐genomes of the DW and *Ae. tauschii* parents and the respective SHW in our study is comparable to modeling GCA and SCA. Both are fundamental sources for prediction based on either pedigree or marker information of the lines forming the interspecific hybridization (SHW).

**TABLE 2:** Variance components for models pedigree model (PM), marker model (GM), pedigree + marker model 1 (PGM‐1), PGM‐2, PGM‐3 for all diseases traits, tan spot (TS), septoria nodorum blotch (SNB), and spot blotch (SB).

		Variance components								
Trait	Model	$\sigma_{d_{p}}^{2}$	$\sigma_{t_{I}}^{2}$	$\sigma_{c_{p}}^{2}$	$\sigma_{c_{m}}^{2}$	$\sigma_{H_{d}}^{2}$	$\sigma_{H_{t}}^{2}$	$\sigma_{H_{c}}^{2}$	$\sigma_{H_{dxt}}^{2}$	$\sigma_{e}^{2}$
	PM	0.254	0.197	0.281						0.424
TS	GM				0.555					0.330
	PGM‐1	0.121	0.096	0.109	0.412					0.213
	PGM‐2					0.192	0.120	0.453		0.265
	PGM‐3							0.497	0.207	0.252
	PM	0.220	0.265	0.194						0.453
SNB	GM				0.727					0.403
	PGM‐1	0.118	0.129	0.100	0.542					0.280
	PGM‐2					0.179	0.134	0.594		0.342
	PGM‐3							0.668	0.213	0.325
	PM	0.190	0.211	0.184						0.582
SB	GM				0.604					0.536
	PGM‐1	0.134	0.138	0.124	0.362					0.395
	PGM‐2					0.228	0.130	0.438		0.472
	PGM‐3							0.534	0.204	0.473

Similar proportions of variance were explained by the DW and *Ae. tauschii* parents ($\mathrm{GCA}:\sigma_{d_{p}}^{2},\;\sigma_{t_{I}}^{2}$) and of the SHW ($\mathrm{SCA}:\;\sigma_{c_{p}}^{2})$ for all traits using the PM model (Table 2). For GM, PMG‐1, PGM‐2, and PGM‐3, a much larger proportion of variance was explained by genomic SCA $(\;\sigma_{c_{m}}^{2}=0.727\;\mathrm{for}\;\mathrm{GM},\;\;\sigma_{c_{m}}^{2}=0.542\;\mathrm{for}\;\mathrm{PGM}-1,\;\sigma_{c_{m}}^{2}=0.594\;\mathrm{for}\;\mathrm{PGM}-2,\;\;\mathrm{and}\;\sigma_{H_{c}}^{2}$ = 0.668 for PGM‐3). Trends for all traits were similar.

The general higher proportion of variance by SCA indicates that dominance and epistasis play a large role in a situation of allopolyploidization. Epistatic interaction between the A, B, and D genomes was also already suggested for TS resistance by Lozano‐Ramirez et al. (2022a). Genomic loci from all three genomes were contributing to the resistance in the SHW while the DW parents showed moderately susceptible to susceptible reaction types, suggesting possible favorable epistatic interaction (activation) between genomes. Epistatic interaction activating or suppressing disease resistance have also been reported in other studies on wheat (Chen et al., 2023; Chu et al., 2008; Hiebert et al., 2020; Nelson et al., 1997). Genes may be activated or suppressed in SHW after hybridization. For instance, the resistance to stem rust pathogen (*Puccinia graminis *f. sp.* tritici*) is suppressed in the hexaploid state by Med15, a component of the Mediator complex encoded by the D sub‐genome (Hiebert et al., 2020). These findings are also aligned with the observed alternations in gene expression during allopolyploidization (Kenan‐Eichler et al., 2011; Li et al., 2014; Vasudevan et al., 2023; M. Yu et al., 2017). Early research studies documented that the onset of both allotetra‐ and allohexa‐polyploidization events in wheat is associated with rapid and extensive structural changes at the genetic level (Ozkan et al., 2001; Shaked et al., 2001), as well as the gene expression and/or epigenetic level in wheat (Adams & Wendel et al., 2005). However, more recent work suggested that most structural changes may have taken place during allotetraploidization (Mestiri et al., 2010; Zhang et al., 2013). Compared with their parental lines, changes in genome structures and copy numbers of certain genes have been observed in SHW (Zhao et al., 2011; Zhao et al., 2011), while once SHW archive euploid chromosome sets, they are genetically stable (Mestiri et al., 2010; Zhao et al., 2011). The possible changes in genome structure during allohexaploidization indicate that pedigree information might not be the best predictor for crosses forming the SHW. Furthermore, some genetic variation remaining within *Ae. tauschii* accessions derived from gene banks is likely, and thus the identity of each accession might not always reflect the same genotype in a cross.

In addition to the early reports of gene expression and epigenetic changes, Rapp et al. (2009) defined the concept of “expression‐level dominance” evaluating gene expression patterns during allopolyploidization in cotton. Expression‐level dominance means that the total expression level for all homeologs of a gene in a polyploid is statistically equivalent to the expression level of one of the polyploid parents instead of additivity between homoeologs. Such a pattern suggests that there has been sub‐functionalization of genes from the two parental lines to some extent in SHW. Li et al. (2014) identified functional dominance of genes in SHW with similar expression levels to those in tetraploid parent enriched for plant development, and genes with similar expression levels to those in *Ae. tauschii* were found to be involved in adaptation. Expression‐level dominance in SHW could contribute to SCA variance.

### 3.2 Genomic prediction

It has been proven in wheat (Crossa et al., 2007; Pérez‐Rodríguez et al., 2012) that large linkage blocks allow for relatively stable epistatic effects within and between the sub‐genomes A, B, and D. The use of nonlinear kernels such as the GK enables the capturing of cryptic small epistatic effects. In this study of SHW, the better predictive models were mostly those using the nonlinear GK that is supposed to capture gene × gene interaction, especially those existing between sub‐genomes AB and D.

To our knowledge, this is the first study that uses genomic models developed for hybrid breeding to predict the phenotypes of allopolyploids as a hybrid of different species. The prediction problems applied in this study are graphically displayed in Figure 3. The three examples of CV (CV1, CV2, and CV3) represent major problems CIMMYT pre‐breeders and breeders face when testing SHW lines for any agronomic trait or when planning a new generation of SHW lines. CV1 predicts the performance of new SHW, based on the subset of SHW evaluated in the field, while other SHW are unobserved (missing). CV1 mirrors the problem, not having the full capacity to evaluate all available SHW phenotypically in the field or in the greenhouse, especially for time‐consuming and cost‐intensive phenotypes. CV2 and CV3 perform CV on the DW and *Ae. tauschii* parents, respectively. If a set of DW or *Ae. tauschii* parents were assigned to training and testing sets, all the corresponding SHW synthesized with these parents were also assigned to training‐testing sets. For example, the training set includes all the SHW obtained when using 80% of the DW parents crossed with any *Ae. tauschii* accession and predicting the remaining 20% of the SHW. Both prediction problems, CV2 and CV3, mimic the problem of predicting SHW using parents (DW or *Ae. tauschii* accessions) that were not necessarily tested in combination.

**FIGURE 4:**
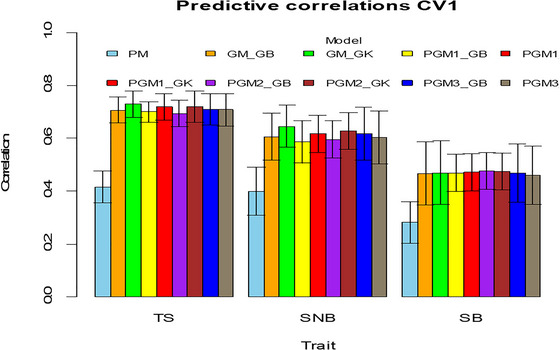
Average Pearson correlation (bars) and standard deviation (whiskers) between observed and predicted values for fivefold random cross‐validation (CV) for disease traits tan spot (TS), septoria nodorum blotch (SNB), and spot blotch (SB) for the prediction of crosses (CV1) for the PM, GM.GB, GM.GK, PGM‐1.GB, PGM‐1.GK, PGM‐2.GB, PGM‐2.GK, PGM‐3.GB, and PGM‐3.GK models.

**FIGURE 5:**
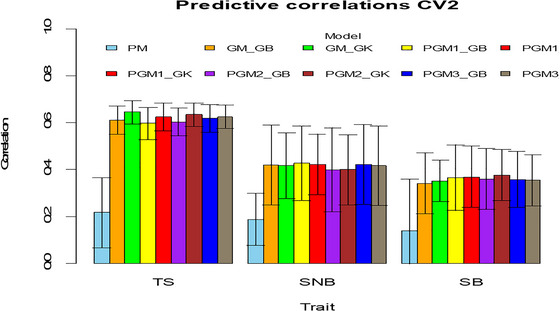
Average Pearson correlation (bars) and standard deviation (whiskers) between observed and predicted values for fivefold random cross‐validation (CV) for disease traits tan spot (TS), septoria nodorum blotch (SNB), and spot blotch (SB) for the prediction of crosses (CV2) for the PM, GM.GB, GM.GK, PGM‐1.GB, PGM‐1.GK, PGM‐2.GB, PGM‐2.GK, PGM‐3.GB, and PGM‐3.GK models.

**FIGURE 6:**
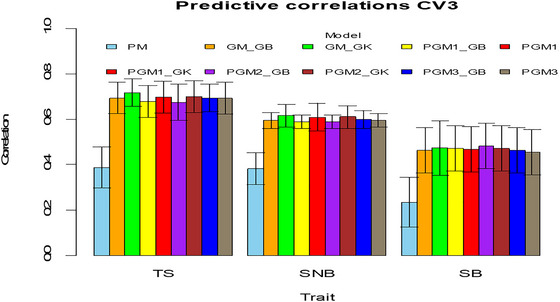
Average Pearson correlation (bars) and standard deviation (whiskers) between observed and predicted values for fivefold random cross‐validation (CV) for disease traits tan spot (TS), septoria nodorum blotch (SNB), and spot blotch (SB) for the prediction of crosses (CV3) for the PM, GM.GB, GM.GK, PGM‐1.GB, PGM‐1.GK, PGM‐2.GB, PGM‐2.GK, PGM‐3.GB, PGM‐3.GK models.

The prediction abilities obtained varied with correlations between the observed and predicted values. These differences were caused by the model applied and the individual prediction problems tested (Table 3 and Figure 4 for random cross‐validation CV1 scheme, Table 4 and Figure 5 for random cross‐validation CV2 scheme, and Table 5 and Figure 6 for random cross‐validation CV3). In general, for all three testing schemes, it was found that the predictive ability was the lowest when PM was used and slightly increasing when using GK compared to GB. The highest prediction abilities for CV1 were achieved with GK models; for example, the highest correlations were 0.730 (GM‐GK) and 0.720 (PGM‐2‐GK) for TS and 0.645 (GM‐GK) for SNB (Table 3 and Figure 4). Prediction abilities were generally lower for models PM and PGM‐3.

**TABLE 3:** Average Pearson correlation between observed and predicted values for fivefold random cross‐validation validation for prediction from missing crosses (CV1) for disease traits tan spot (TS), septoria nodorum blotch (SNB), and spot blotch (SB). For the pedigree model (PM), marker model (GM), pedigree + marker model 1 (PGM‐1), PGM‐2, and PGM‐3 models.

		Models								
Traits		PM	GM		PGM‐1		PGM‐2		PGM‐3	
		A	GB	GK	GB	GK	GB	GK	GB	GK
TS	Mean	0.416	0.707	**0.730**	0.700	0.719	0.693	0.720	0.709	0.708
	SD	0.06	0.05	0.05	0.04	0.05	0.05	0.06	0.06	0.06
SNB	Mean	0.399	0.606	**0.645**	0.587	0.617	0.596	0.629	0.618	0.603
	SD	0.09	0.09	0.08	0.08	0.07	0.07	0.07	0.10	0.10
SB	Mean	0.282	0.467	0.470	0.468	0.471	**0.477**	0.473	0.469	0.461
	SD	0.08	0.12	0.12	0.07	0.07	0.07	0.07	0.11	0.11

*Note*: Models with the best genomic prediction ability for each trait are shown in bold.

Abbreviations: GB, GBLUP matrix; GK, Gaussian kernel matrix.

**TABLE 4:** Average Pearson correlation between observed and predicted values for fivefold random cross‐validation for prediction from missing durum wheat (DW) parents (CV2) for disease traits tan spot (TS), septoria nodorum blotch (SNB), and spot blotch (SB). For the pedigree model (PM), marker model (GM), pedigree + marker model 1 (PGM‐1), PGM‐2, and PGM‐3 models.

		Models								
Traits		PM	GM		PGM‐1		PGM‐2		PGM‐3	
		A	GB	GK	GB	GK	GB	GK	GB	GK
TS	Mean	0.217	0.612	**0.646**	0.597	0.626	0.603	0.635	0.620	0.626
	SD	0.15	0.06	0.05	0.07	0.06	0.06	0.05	0.06	0.05
SNB	Mean	0.188	0.421	0.417	**0.428**	0.423	0.399	0.401	0.423	0.417
	SD	0.11	0.17	0.14	0.16	0.13	0.18	0.15	0.17	0.17
SB	Mean	0.140	0.342	0.353	0.367	0.370	0.360	**0.378**	0.359	0.354
	SD	0.22	0.13	0.09	0.14	0.13	0.13	0.11	0.12	0.11

*Note*: Models with the best genomic prediction ability for each trait are shown in bold.

Abbreviations: A, pedigree matrix; GB, GBLUP matrix; GK, Gaussian kernel matrix.

**TABLE 5:** Average Pearson correlation between observed and predicted values for fivefold random cross‐validation for prediction from missing *Ae. tauschii* parents (CV3) for disease traits tan spot (TS), septoria nodorum blotch (SNB), and spot blotch (SB). For the pedigree model (PM), marker model (GM), pedigree + marker model 1 (PGM‐1), PGM‐2, and PGM‐3 models.

		Models								
Traits		PM	GM		PGM‐1		PGM‐2		PGM‐3	
		A	GB	GK	GB	GK	GB	GK	GB	GK
TS	Mean	0.389	0.694	**0.718**	0.680	0.699	0.675	0.702	0.694	0.692
	SD	0.09	0.07	0.06	0.07	0.07	0.08	0.07	0.06	0.07
SNB	Mean	0.382	0.596	**0.617**	0.591	0.610	0.591	0.611	0.600	0.596
	SD	0.071	0.036	0.05	0.03	0.06	0.03	0.05	0.04	0.03
SB	Mean	0.235	0.463	0.473	0.472	0.468	**0.483**	0.472	0.464	0.454
	SD	0.11	0.10	0.12	0.10	0.10	0.10	0.10	0.10	0.10

*Note*: Models with the best genomic prediction ability for each trait are shown in bold.

Abbreviations: A, pedigree matrix; GB, GBLUP matrix; GK, Gaussian kernel matrix.

The highest prediction abilities for CV2 were also obtained with GK, except for the model PGM‐1 for trait SNB (0.428) (Table 4). Similarly, the GK resulted in slightly higher prediction abilities for all the traits except for SB for cross‐validation CV3 with prediction accuracies of 0.483 for traits with the model PGM‐2 (Table 5).

While the models including SNP marker data showed no significant differences, the GM and PGM‐2 models showed slightly higher prediction abilities. The PM model revealed the lowest prediction ability for all diseases, up to twofold. Furthermore, prediction abilities tend to be higher for CV1 than for CV2 (Table 4, Figure 5) and CV3 (Table 5, Figure 6), regardless of the model deployed. Higher prediction abilities were observed for CV3, which assigned the *Ae. tauschii* parent into folds, compared to CV2 for almost all the models and traits. A much larger number of *Ae. tauschii* accessions were used for the synthesis of SHW compared to a smaller number of DW parents, resulting in more crosses made per DW parent. A favorable genetic component due to any closer relationship between the fewer DW parents (including sister lines) was therefore not apparent in CV2.

Across all predictions, the standard deviations of the predictive correlations are relatively large, leading to the PM model being the only model with significantly lower results. Overall, levels of prediction abilities for the diseases presented were similar or higher in comparison to previous studies. SB was the trait with the lowest prediction ability. Daetwyler et al. (2014) predicted leaf rust resistance in a set of diverse bread wheat landraces and reported average prediction abilities of 0.35. More recently, Semagn et al. (2022) described prediction accuracies for TS with values between 0.11 and 0.41 in an association mapping panel of Canadian spring wheat cultivar within and among environments. Our results were also comparable to those that explore predictions of grain yield in hybrid wheat (Basnet et al., 2019; Y. Zhao et al., 2013). In general, CV schemes predicting the performance of untested hybrids, equivalent to the prediction of the performance of untested SHW (CV1) in our study, showed better results than CV schemes predicting untested female or male parents.

In general, when using the nonlinear kernel (GK), in the GM, PGM‐1, PGM‐2, PGM‐3 models, the prediction average consistently increases compared to the use of linear GB (Tables 3 **–** 5, Figures 4 **–** 6) for all diseases but is more noticeable in the SNB. This result is consistent with what was previously discussed regarding SCA manifesting nonadditive effects (epistasis) that can be captured better by the nonlinear kernel (GK) than by the linear kernel (GB). Clearly, the PM is overcome, in terms of prediction accuracy, by all the other model‐kernel types. Genomic prediction for models and kernel type seems to be different for the different diseases investigated in this study. For example, for TS and SNB diseases, the best model‐kernel options were GM‐GK and PGM‐3‐GK. For the disease SB, models PMG‐1 and PMG‐2 are slightly superior in genomic prediction ability to the others yet with few differences between kernel types.

The CIMMYT produced ∼1600 SHW since the 1980s and subsequently generated thousands of crosses with elite bread wheat. Synthetic derivative lines have been selected as parents for mainstream breeding, with rigorous selection resulting in advanced lines with excellent performance for yield and other traits. Synthetic derivative lines have been selected as candidates in international yield trials, which are disseminated by the CIMMYT globally every year (Dreisigacker et al., 2008; Rosyara et al., 2019). Many of the CIMMYT SHW lines have been evaluated by different research groups and for a series of diverse traits including diseases, quality characteristics, and agronomic traits. However, only smaller numbers of subsets of SHW that allow manageable field or greenhouse trials have been performed. Our results therefore provide a new approach to guide breeders assessing additional untested SHW.

### 3.3 Model comparison

The results of the hypothesis tests for the mean differences between model PM and each of the GM, PGM‐1, PGM‐2, PGM‐3 models indicate that the differences in their means are significant at the 0.05 level. However, when comparing the means of GM with the mean of each of the PGM‐1, PGM‐2, PGM‐3 models, it was found that the differences in their means are not significant, despite some noticeable differences in the means of PGM‐3 compared to the other models, as observed in the graphs. The contribution of genomic information from the parents in prediction appears to be positive in terms of increasing the predictions, although this increase does not meet the levels of statistical significance. This is likely due to the lack of information about the markers of the parents, and in the case of *Ae. tauschii*, there were 127 markers available out of a total of 264, and the pedigree matrix was not available (see Table 1).

## 4 CONCLUSION

The genomic prediction approach for this type of material is the main novelty of this study. We could draw sound and robust conclusions despite a limited phenotype data. We successfully applied the quantitative genetics tools, originally developed for the prediction of intraspecific hybrids to a situation of allopolyploidization. We predicted the phenotypes of SHW for four major quantitatively inherited diseases in wheat. Our study describes a predictive approach that could further guide the use of genetic resources available in gene banks to increase the extent of useful genetic diversity available to breeders. Results indicated that the genomic prediction of several wheat diseases in the SHW developed from the cross of DW with *Ae. tauschii* can be successfully achieved. In general, genomic prediction models including pedigree and nonlinear kernels overcome the prediction ability of genomic models that use only linear kernels. These results indicated that nonlinear kernels explained and predicted the complex genome interaction existing when crossing DW and *Ae. tauschii* better than the linear kernel.

## AUTHOR CONTRIBUTIONS

**Susanne Dreisigacker**: Conceptualization; investigation; methodology; project administration; supervision; validation; writing—original draft; writing—review and editing. **Johannes Martini**: Conceptualization; investigation; visualization; writing—original draft; writing—review and editing. **Jaime Cuevas**: Data curation; formal analysis; investigation; writing—original draft. **Paulino Perez‐Rodriguez**: Data curation; formal analysis; investigation; methodology. **Nerida Lozano Ramirez**: Data curation; investigation; resources; validation; visualization; writing—original draft. **Julio Huerta‐Espino**: Data curation; funding acquisition; investigation; project administration. **Pawan Kumar Singh**: Data curation; investigation; methodology; project administration. **Leonardo Crespo**: Conceptualization; data curation; investigation. **Alison Bentley**: Conceptualization; investigation; methodology; supervision; validation. **Jose Crossa**: Conceptualization; investigation; methodology; writing—original draft; writing—review and editing.

## CONFLICT OF INTEREST STATEMENT

The authors declare no conflicts of interest.

## Supporting information


**Supplementary Table S1**: Synthetic hexaploid wheat (SHW) lines with their DW and *Ae. tauschii* parents.

## Data Availability

The phenotypic (TS, SNB and SB) and genotypic data sets can be found in the following link: https://hdl.handle.net/11529/10548948.
